# Patient preferences for maintenance therapy in Crohn’s disease: A discrete-choice experiment

**DOI:** 10.1371/journal.pone.0227635

**Published:** 2020-01-16

**Authors:** Glen S. Hazlewood, Gyanendra Pokharel, Robert Deardon, Deborah A. Marshall, Claire Bombardier, George Tomlinson, Christopher Ma, Cynthia H. Seow, Remo Panaccione, Gilaad G. Kaplan

**Affiliations:** 1 Department of Medicine and Community Health Sciences, Cumming School of Medicine, University of Calgary, Calgary, Alberta, Canada; 2 Department of Mathematics and Statistics, University of Calgary, Calgary, Alberta, Canada; 3 Department of Production of Animal Health, Faculty of Veterinary Medicine, University of Calgary, Calgary, Alberta, Canada; 4 Department of Medicine and Institute of Health Policy, Management, and Evaluation, University of Toronto, Toronto, Ontario, Canada; 5 Toronto General Research Institute, University Health Network, Toronto, Ontario, Canada; 6 Inflammatory Bowel Disease Unit, Division of Gastroenterology and Hepatology, Cumming School of Medicine, University of Calgary, Calgary, Alberta, Canada; London School of Hygiene and Tropical Medicine, UNITED KINGDOM

## Abstract

**Objective:**

To quantify patient preferences for maintenance therapy of Crohn’s disease and understand the impact on treatment selection.

**Methods:**

We conducted a discrete-choice experiment in patients with Crohn’s disease (n = 155) to measure the importance of attributes relevant to choosing between different medical therapies for maintenance of Crohn’s disease. The attributes included efficacy and withdrawals due to adverse events, as well as dosing and other rare risks of treatment. From the discrete-choice experiment we estimated the part-worth (importance) of each attribute level, and explored preference heterogeneity through latent class analysis. We then used the part-worths to apply weights across each outcome from a prior network meta-analysis to estimate patients’ preferred treatment in pairwise comparisons and for the overall group of treatments.

**Results:**

The discrete-choice experiment revealed that maintaining remission was the most important attribute. Patients would accept a rare risk of infection or cancer for a 14% absolute increased chance of remission. Latent class analysis demonstrated that 45% of the cohort was risk averse, either to adverse events or requiring a course of prednisone. When these preferences were used in modelling studies to compare pairs of treatments, there was a ≥ 78% probability that all biologic treatments were preferred to azathioprine and methotrexate, based on the balance of benefits and harms. When comparing all treatments, adalimumab was preferred by 53% of patients, who were motivated by efficacy, and vedolizumab was preferred by 30% who were driven by the preference to avoid risks. However, amongst biologic treatment options, there was considerable uncertainty regarding the preferred treatment at the individual patient level.

**Conclusion:**

Patients with Crohn’s disease from our population were, on average, focused on the benefits of treatment, supporting intensive treatment approaches aimed at maintaining remission. Important preference heterogeneity was identified, however, highlighting the importance of shared decision making when selecting treatments.

## Introduction

Crohn’s disease is a complex chronic inflammatory disorder with rising prevalence [1–4]. Crohn’s disease is treated pharmacologically with immunosuppressants and/or biologics, with the aim of achieving clinical and endoscopic remission [5]. Over the past two decades, the therapeutic armamentarium for Crohn’s disease management has expanded dramatically, particularly with the introduction of biologic agents [6]. Choosing between these treatments is challenging because patients and physicians must balance trade-offs between efficacy, potential toxicity and other considerations such as route of administration [7]. In a shared decision-making model, health care providers work with patients to help them understand the trade-offs and their own preferences, and to choose the best treatment.

A discrete-choice experiment (DCE) is an experimentally designed survey in which participants are asked to state their preferred treatments across a series of survey questions where they are presented with a choice between 2 or more hypothetical options [8, 9]. The treatments are described in terms of properties (attributes), each of which has different possible quantitative or qualitative values (levels). By analyzing patients’ responses across a series of designed choice tasks, the relative importance of each attribute level can be quantified. This information can be useful in understanding how to weigh competing risks and benefits within treatment recommendations or in informing decision aids for patients that promote shared decision-making. Additionally, through latent class models, heterogeneity in preferences can be explored, which can help understand the diversity in patient preferences.

The objective of this study was to quantify patient preferences for maintenance therapy in Crohn’s disease and explore preference heterogeneity. We also sought to demonstrate how the preference information can be integrated with efficacy and safety data derived from a network meta-analysis (NMA) to estimate the preferred treatment(s).

## Methods

### Design of discrete-choice experiment

#### Selection of attributes and levels

The DCE was developed in an iterative process following published guidance [9]. The selection of attributes and levels were guided from our research question. We were interested in understanding patient preferences for key considerations that would be discussed with patients in an evidence-informed discussion. As such, the attributes included common outcomes measured in randomized trials that could be presented to patients in a decision-aid, informed from comparative effectiveness research. We used the primary benefit and toxicity attributes from our published NMA [10]. This included maintenance of remission at trial end (typically measured in clinical trials as Crohn’s disease activity index score less than 150 at 1-year) and adverse events leading to discontinuation of the medication (measured as withdrawals due to adverse events in clinical trials). Additional attributes included other considerations not captured as outcomes in clinical trials, but relevant to an evidence-informed discussion of common treatment options for maintenance therapy: dosing of the medication (use of a daily oral pill, weekly or biweekly injection, intravenous infusion or a combination of infusion and pill); possible low blood counts or liver reaction; need for a course of prednisone; and small risk of serious infection and possible increased risk of certain cancers (Table 1).

**Table 1:** Attributes and levels for the discrete-choice experiment.

Attribute	Levels
**NMA outcomes**	
Chance of remission (for at least one year)	20 out of 100 people
	50 out of 100 people
	80 out of 100 people
Chance of having a side effect that requires you to stop the medication	1 out of 100 people
	15 out of 100 people
	30 out of 100 people
**Other properties of treatments**	
How you take the medication(s)	One medication: Daily tablets
	One medication: Tablets twice a day
	One medication: Injection at home every week
	One medication: Injection at home every 2 weeks
	One medication: Intravenous infusion in a clinic or hospital every 8 weeks
	Two medications: Daily tablets and Intravenous infusion in a clinic or hospital every 8 weeks
Take a short course of prednisone in addition to other medications	Yes
	No
Possible low blood counts or liver reaction	Yes
	No
Small risk of serious infections and possible increased risk of certain cancers	Yes
	No

NMA, network meta-analysis

#### Construction of choice tasks

Participants completed a series of 13 choice tasks in which they chose between 1 of 3 treatment options. The choice tasks were constructed using a balanced overlap, fractional factorial design in Sawtooth Software (Orem, USA). This design follows best practices for experimental designs (e.g. orthogonality), but intentionally includes some overlap in the levels between choices. While this sacrifices the efficiency of the DCE somewhat (reducing the precision around the estimates), it helps reduce the complexity of each task, as patients have to consider fewer trade-offs. We found this helpful in a previous DCE study [11], and in pre-testing with this survey. Using the balanced overlap design, we generated 100 different versions of the 13 choice sets, and randomly distributed these to the patients. Using 100 (or more) survey versions has been recommended for randomized designs to ensure optimal coverage of the potential design space [12].

#### Survey design

The design of the survey was modeled after a previous DCE study [11]. The survey included a series of lead-in screens to explain each attribute and the task required. Prior to the 13 choice tasks, patients also completed 2 warm-up questions with simplified trade-offs to help introduce the tasks. Each warm-up also included a dominated alternative, where one treatment was better across all attributes. This served as a measure of internal validity. We refined a draft survey through iterative pre-testing, where a trained research assistant conducted 1-on-1 interviews with eligible patients to improve the clarity and usability of the computer-based survey. Pre-testing ended when no further issues were identified (n = 6). Screenshots of the final survey are presented in the Supplementary Material (S1 Fig).

#### Administration

Adult (age>18 years) patients with Crohn’s disease were recruited from outpatient clinics in Calgary, AB between July 2016 and November 2016. All patients had a comprehensive chart review to confirm diagnosis based on Lennard Jones Criteria [13], and to extract clinical information including: age at diagnosis; disease duration; disease location; disease behavior; perianal disease; prior exposure to immunosuppressants, corticosteroids and biologics; and prior intestinal resection. Patients had the option of completing the survey in clinic on a laptop computer or online at home. Patients who chose to complete the survey at home received reminders at 1, 2, and 4 weeks if they had not completed the survey. The survey was hosted using Sawtooth Software (Orem, USA) on a secure server. This study was conducted according to the principles outlined in the Declaration of Helsinki with informed consent obtained from all patients. The study was approved by the University of Calgary Conjoint Health Research Ethics Board (#REB15-1677).

### Analysis

#### Primary analysis

From the DCE, we estimated the importance (utility) of each attribute level using a hierarchical Bayesian model. As with other analytic approaches, this model assumes that in each choice task, participants select the option with the highest overall utility. The utility is not observed, but is modeled as a latent variable as a function of the attribute levels that define it. In the hierarchical Bayesian model, each participant’s utility function is assumed to vary randomly around a common mean that is shared across participants. The model was fitted using published code (see Supplementary Material, S1 Text) [14]. The 2 trial attributes ‘chance of remission’ and ‘chance of adverse events requiring stopping the medication’ were modeled on a linear scale, after confirming a linear relationship across the range of levels evaluated.

The part-utility values are not meaningful in isolation, but can be used to compare the relative importance of attributes. To aid in interpretation, we scaled the values from -10 (strong aversion) to +10 (strong preference). We also calculated both the relative importance (RI) and marginal rate of substitution (MRS). The RI was calculated by scaling the overall importance of each attribute (difference between levels with highest and lowest values) such that the sum across all attributes totaled 100. The MRS was calculated as the increase in ‘chance of remission’ patients would require to accept each undesirable attribute. The 95% credible intervals (CrI) of the posterior predictive distribution of the MRS were estimated using Monte Carlo samples of the parameters from the posterior distribution.

As measures of internal validity, we determined the percent of patients who failed one or both of the dominated alternative warm-up tasks. We also compared the distribution of responses across the 3 options (rightmost option, middle, leftmost option) to explore whether there was any evidence of ‘straight-lining’, where a patient chose the same option in all (or nearly all) tasks [15]. Finally, we also calculated the frequency of attribute dominance, where a participant always chose the option with the ‘best’ level for a certain attribute.

The analyses were performed using R statistical software version 3.3.1 with rjags package version 4–6 (www.r-project.org) running JAGS 4.2.0. Minimally informative prior probability distributions were used for all parameters. Convergence was assessed by running 3 Markov Chain Monte Carlo (MCMC) chains, inspecting the sampling history plots and calculating Gelman–Rubin–Brooke (GBR) statistics [16]. The GBR statistics rely on parallel chains to test whether they all converge to the same posterior distribution. This method essentially measures whether there is a significant difference between the variance within and between several MCMC chains by a statistic called *scale reduction factor*. The chains are converged when the variation between the chain is very small so that the estimate of the scale reduction factor is close to 1, with a threshold of <1.10 commonly recommended [16]. We used 350,000 monitoring iterations in each of 3 chains after 150,000 burn-in iterations.

#### Latent class analysis

To explore preference heterogeneity, we conducted latent class analyses. A latent class analysis identifies groups with similar preferences, and estimates the probability each patient belongs to each class. We presented results for both the 2-group and 3-group solutions; solutions with more than 3-groups had a worse measure of model fit (Bayesian information criteria). We conducted logistic regression models on the 2-group solution to determine the association between patient characteristics and latent class membership. Patients were assumed to belong to the class for which they had the highest probability. Latent class analyses were conducted using SSI Web version 8.3.6 (Sawtooth Software) and regression models in R (version 3.3.1; http://www.r-project.org).

#### Exploratory treatment scenario analyses

To illustrate how patient preferences may impact treatment selection we conducted modelling studies. In these analyses, common with DCEs, the part-worths of the various attributes that define a treatment are added to estimate the overall ‘value’ of a treatment. While not meaningful in isolation, the overall value of 2 or more treatments can be compared to estimate which treatment patients are likely to prefer, based on the balance of benefits and harms. The attribute levels assigned to each treatment are presented in Table 2. The estimates of treatment benefit (maintenance of remission) and adverse events (withdrawals due to adverse events, WDAE) were derived from a prior NMA of maintenance options for Crohn’s disease [10]. We used the outcome maintenance of remission from the NMA (as opposed to induction of remission that was also reported in the NMA), as this corresponded to our DCE attribute of staying in remission for at least one year. The treatments evaluated in this NMA included both immunomodulators and biologics: methotrexate, azathioprine, infliximab, infliximab + azathioprine, adalimumab and vedolizumab. For each treatment, we calculated the absolute risk for both outcomes by multiplying the treatment effect (odds ratio) relative to placebo by the assumed baseline value for the placebo group, which was estimated as the median value from a Bayesian random effect model of the placebo arms of all trials in the NMA.

**Table 2:** Treatment trade-offs.

Attribute	Treatment					
	Infliximab	Infliximab + azathioprine	Adalimumab	Vedolizumab	Azathioprine	Methotrexate
Probability of maintaining remission at 1 year from NMA, median (95% CrI)	48% (33 to 64)	63% (44 to 80)	61% (46 to 75)	41% (27 to 57)	36% (24 to 49)	42% (25 to 61)
Probability of withdrawal due to adverse events at 6 months from NMA, median (95% CrI)	12% (6.3 to 25)	12% (4.5 to 27)	2.3% (1.2 to 4.2)	3.0% (1.4 to 6.1)	15% (7.9 to 26)	38% (12 to 83)
Dosing regime	Intravenous infusions every 8 weeks	Intravenous infusions every 8 weeks + daily pills	Subcutaneous injections every 2 weeks	Intravenous infusions every 8 weeks	Daily pills	Weekly injections
Short course of prednisone initially	Yes†	Yes†	Yes†	Yes†	Yes	Yes
Small increased risk of serious infection and possible increased risk of certain cancers	Yes	Yes	Yes	No	Yes	Yes
Possible low blood counts or liver reaction	No††	Yes	No††	No	Yes	Yes

†Varied in a sensitivity analysis to ‘No’

††Varied in a sensitivity analysis to ‘Yes’

NMA, network meta-analysis; CrI, credible interval

In the modelling studies, patients were assumed to choose the treatment with the highest overall value (utility), which was calculated as a sum of the part-utilities of the attributes. To account for the variability in preferences and the imprecision in both the NMA and DCE estimates, we conducted 10,000 analyses for each patient, sampling the NMA outcomes and part-worths from their Bayesian posterior distributions [14]. The results were averaged across all draws and reported as the probability that patients would choose each treatment option. We modelled a choice between all 6 treatment options in pair-wise comparisons and as an overall group. We conducted a sensitivity analysis, where biologic therapy was given without a course of prednisone, as prednisone can be avoided in some circumstances due to the more rapid onset of activity (<6–8 weeks) as compared to immunomodulators (>8–12 weeks). We also included a sensitivity analysis where both infliximab and adalimumab were assumed to have a risk of “low blood counts or liver reaction” as both have been described with anti-TNF therapy in rare cases [17, 18], albeit with considerably lower frequency than with azathioprine or methotrexate [19].

## Results

### Patient characteristics

The demographics and disease characteristics of the 155 patients who completed the survey are presented in Table 3. Disease characteristics were similar to Crohn’s disease patients followed in adult GI clinical practice [20]. The median age was 40, 68% were female, and the median disease duration was 10 years. Nearly half (48%) had ileocolonic disease, and 41% had had prior surgery. Most patients had been treated with corticosteroids (61%) and azathioprine (79%) and many had received anti-Tumour Necrosis Factor (TNF) therapy; either infliximab (45%) or adalimumab (50%). Few patients (4%) had been treated with vedolizumab.

**Table 3:** Patient characteristics.

Characteristic		Value
**Demographics and disease characteristics**		
Age, years, median (IQR)		40 (22)
Age at diagnosis, years, n (%)		
	< 16	15 (9.7)
	16–40	116 (74.8)
	> 40	24 (15.5)
Female, n (%)		106 (68.4)
Current or former smoker, n (%)		38 (24.5)
Disease duration, years, median (IQR)		10 (15.0)
Location of Crohn’s Disease		
	Ileal	38 (24.5)
	Colonic	43 (27.7)
	Ileocolonic	74 (47.7)
Perianal disease, n (%)		37 (23.8)
Prior abdominal surgery, n (%)		63 (40.7)
Disease behavior, n (%)		
	Fibrostenosis	30 (19.4)
	Inflammation	85 (54.8)
	Penetrating	40 (25.8)
**Prior treatment**		
Azathioprine, n (%)		123 (79.4)
Corticosteroids, n (%)		95 (61.3)
Infliximab, n (%)		69 (44.5)
Adalimumab, n (%)		78 (50.3)
Methotrexate, n (%)		48 (31.0)
Vedolizumab, n (%)		6 (3.8)

IQR, interquartile range

### Results from discrete-choice experiment

#### Validity testing

**Fig 1:**
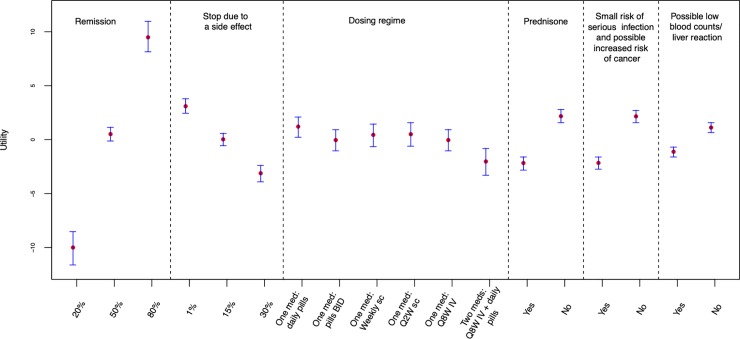
Average importance of each attribute level. Results are presented as utilities [median (95% credible interval)], which are scaled from +10 (strong preference) to– 10 (strong aversion). BID, twice daily; IV, intravenous; med, medication; sc, subcutaneous; Q2W, every 2 weeks; Q8W, every 8 weeks.

The results of the DCE are presented graphically in Fig 1 and in detail in the Supplementary Material (S1 Table). The GBR diagnostics were <1.10 for all parameters, indicating model convergence. The patterns of part-worths were all in the expected direction of effect, suggesting task comprehension (Fig 1) and no patients failed either of the 2 dominated alternative tasks. We did not find evidence of straight-lining. The responses were distributed roughly evenly between the 3 options as would be expected; participants chose the leftmost option 29% of the time, middle 35%, rightmost 36%. No patient chose one option more than 9/13 times (69%). Attribute dominance was observed in 15 patients (9.6%), consistent with the lower end of rates in other published DCE surveys [15]; 2 patients always chose the option that had the best level for remission; 7 patients for avoiding prednisone; 5 for avoiding a rare risk of cancer; and 1 for liver/blood monitoring.

#### Overall results

In the DCE, maintenance of remission was the most important attribute (Fig 1). Across the range of levels considered, maintaining remission was 2.5 times more important than withdrawal due to an adverse event (median utility 10 versus 4.07), the next most important attribute (S1 Table). Other attributes were less important. On average, patients were willing to accept a risk of infection/cancer, a course of prednisone, and possible low blood counts/liver reaction for a treatment that had an absolute increase in the chance of remission of 14.4 (12.5 to 16.3), 13.7 (11.9 to 15.5), and 7.3 (5.9 to 8.7) percentage points, respectively (marginal rate of substitution, S1 Table).

#### Latent class analysis (preference heterogeneity)

**Fig 2:**
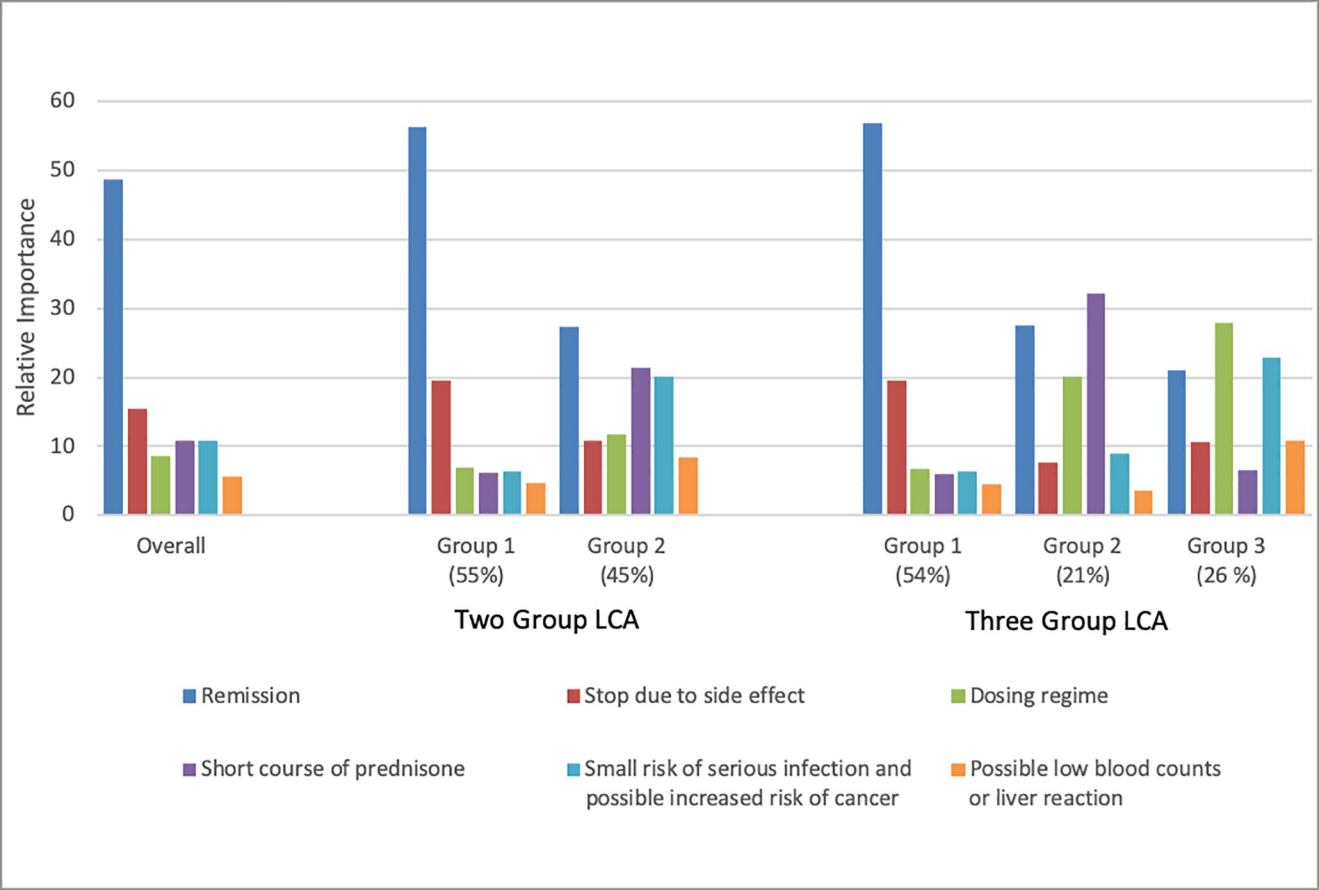
Relative importance of treatment attributes in the overall group and each of the two latent classes. LCA, latent class analysis.

In the 2-group solution for the latent class analysis, most patients (55%) belonged to a group that was highly focused on treatment benefits (Fig 2). The second group was more risk-averse, placing higher importance on avoiding prednisone (relative importance 21% versus 6% in the more risk tolerant group) and avoiding treatments with a risk of cancer/infection (relative importance 20% versus 6% in the more risk tolerant group). In regression models, only prior use of azathioprine was associated with membership in the more risk averse group (OR = 2.60; 95% confidence interval: 1.11, 6.06, S2 Table). In the 3-group solution, the group focused on treatment benefits remained stable with 54% of patients (98% of these were the same patients as in the 2-group solution); however, the risk averse group were further divided into one that had a stronger aversion to prednisone (21% of patients) and another in which dosing and the rare risk of malignancy were the most important attributes (26% of patients). In both the 2 and 3-group solutions, the predictions were quite accurate. In the 2-group solution, only 20/155 (13%) of patients had a probability of group assignment <90%. In the 3-group solution, only 22/155 (14%) of respondents had a probability <90% for their most likely class.

#### Exploratory treatment scenario analyses

When comparing pairs of treatments, there was a ≥78% probability that all biologic-based treatments (infliximab, infliximab + azathioprine, vedolizumab, adalimumab) were preferred to azathioprine and methotrexate, based on the balance of benefits and harms (Table 4). In comparisons between biologic agents, there was a 78% probability adalimumab was preferred to infliximab, and a 73% probability adalimumab was preferred to infliximab + azathioprine. For other pairwise comparisons, the preferred treatment was less certain (Table 4).

**Table 4:** Pairwise treatment comparisons accounting for both NMA outcomes and patient preferences.

	Comparator				
	(probability the intervention is better than the comparator)				
Intervention	Azathioprine	Infliximab	Infliximab + azathioprine	Vedolizumab	Adalimumab
Infliximab	78%	--	--	--	--
Infliximab + azathioprine	80%	53%	--	--	--
Vedolizumab	87%	67%	64%	--	--
Adalimumab	93%	78%	73%	64%	--
Methotrexate	40%	19%	18%	11%	6%

**Fig 3:**
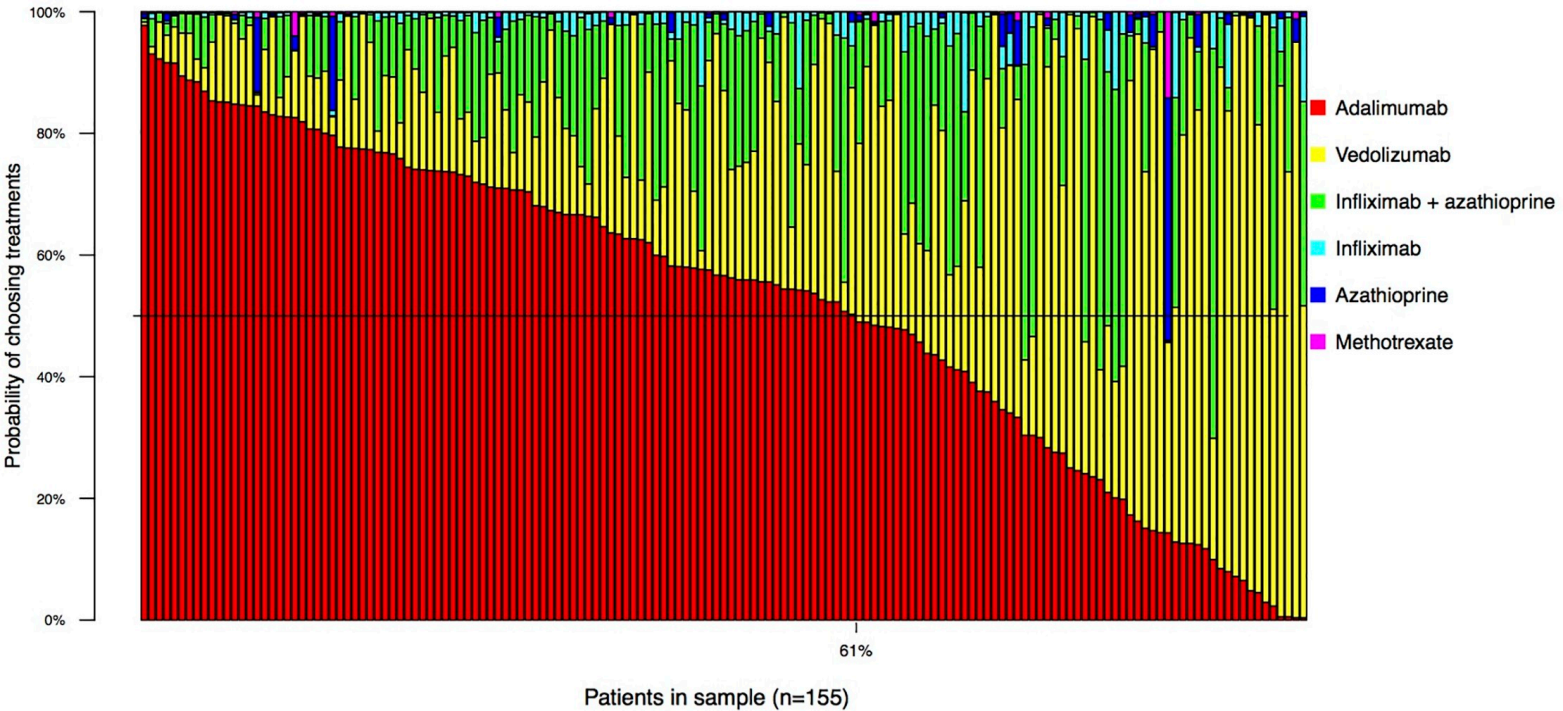
Predicted treatment preferences for each individual patient.

When all treatments were considered together, adalimumab was modelled to be preferred most often (53% of patients), followed by vedolizumab (30%) (S3 Table). The preferences for adalimumab and infliximab + azathioprine were driven by the higher estimates for remission from the NMA, whereas the preference for vedolizumab was driven by the ability to avoid an increased risk of infection/cancer. However, there was considerable uncertainty regarding the preferred treatment at the individual patient level, especially amongst the options adalimumab, vedolizumab, and infliximab + azathioprine (Fig 3).

In the sensitivity analysis where biologic therapy was assumed to not require a course of initial prednisone, biologic therapy was even more preferred to both azathioprine and methotrexate (≥ 92% probability for all pairwise comparisons between biologic therapy and azathioprine or methotrexate, S4 Table). When both infliximab and adalimumab were assumed to also have a risk of “low blood counts or liver reaction”, the strength of preference for both infliximab and adalimumab both decreased somewhat relative to other options (S5 Table). However, when all treatments were considered together, adalimumab was still preferred most often in both sensitivity analyses (53% of patients for the ‘no prednisone’ sensitivity analysis and 42% of patients for the ‘low blood count/liver reaction’ sensitivity analysis, S3 Table).

## Discussion

In this study, we conducted a discrete-choice experiment to understand patient preferences for the trade-offs amongst different maintenance treatments for Crohn’s disease. We found most patients with Crohn’s disease were highly benefit-driven, preferring the use of therapies with the highest likelihood of maintaining remission. In contrast, a smaller group of patients were more risk averse, wishing to minimize potential toxicities, including infection and cancer, even at the expense of reduced likelihood of maintaining remission of Crohn’s disease. Recognizing the heterogeneity of patient preferences among those suffering from Crohn’s disease, physicians can tailor treatment options based on patient preference.

In our analyses, we also applied a novel approach to integrate patient preferences with efficacy and toxicity data from a previously published NMA [10]. The NMA provides the probability of maintaining remission and the likelihood of withdrawing a treatment due to an adverse event. However, judgments are still required to weigh the competing desirable and undesirable aspects of the different treatments. Moreover, patients’ prior experiences may influence their decision-making process when deciding between different attributes of a treatment. For the majority of patients with Crohn’s disease, the primary attribute that influenced their treatment preference was the likelihood of remission, which was highest for adalimumab and infliximab with azathioprine. However, preference of using combination therapy of infliximab and azathioprine was low due to additional medication use and higher risk of toxicity. Among more risk averse patients, the use of gut-selective vedolizumab with a theoretically reduced risk of systemic toxicity was most appealing. While our modelling studies highlighted how considering patient preferences can impact treatment selection, there was considerable uncertainty regarding the preferred treatment amongst biologic options, again illustrating the importance of individualizing these treatment decisions.

The perspective of our study guided the selection and wording of our attributes and levels. We were interested in quantifying patient preferences for key considerations that would be discussed with patients in an evidence-informed shared decision. As such, the benefits and risks had to match the data available from the trials, which imposed restrictions on which attributes were chosen and how they were described. We favoured a text description for the rare risks as opposed to numerical values, which are challenging to communicate and in our experience, rarely done in practice. Our approach is consistent with decision aids that favour simplicity in their presentation [21], and often present rare risks as text descriptors. However, a more in-depth discussion and presentation of treatment risks and benefits may impact patients’ preferences, so this is a potential limitation of our study.

Other studies have evaluated the preferences of patients with Crohn’s disease using a variety of approaches. Our results are consistent with a qualitative study which found that treatment benefits, particularly symptom control, rather than endoscopic remission, are the most important treatment goals [22]. Our findings are also in line with other published DCEs, which have also found that on average, treatment benefits outweigh other considerations for most patients with Crohn’s disease [7, 23–25]. Other studies have also found similar latent classes, with groups of patients that are averse to corticosteroids and rare risks [26, 27]. Our results add to this literature in several regards. First, we provide preference weights to a range of biologic and non-biologic therapy. Second, our sampling in clinic with the linked cohort data allowed us to confirm the diagnoses and include phenotypic data on the patients sampled, not available in most other DCEs in Crohn’s patients to date. Finally, we also demonstrate through our modelling approach, an application of patient preferences against a network meta-analysis for Crohn’s therapies. Other investigators have used modelling approaches, deriving effectiveness estimates from observational data [26]. Using observational data may allow greater flexibility in the choice of attributes (including risks for longer-term outcomes) and modelling approaches, but has the limitation of potential unmeasured confounding when deriving the treatment effect estimates.

Several limitations should be considered for this study. The DCE was conducted in 2016, and we only surveyed patients with Crohn’s disease about treatment options available in Canada at that time. Consequently, certolizumab, natalizumab and ustekinumab were not evaluated. Future studies should evaluate ustekinumab, as it has become a widely used biologic with a separate mechanism of action and a unique, convenient dosing regimen, involving a single intravenous induction dose followed by a self-administered injection every 8 weeks. While our DCE did not capture this dosing regimen, the route of administration was not considered a major factor influencing patient preference in our DCE. Additionally, patient preferences in relation to biosimilars were not considered. Further, we surveyed 155 patients with Crohn’s disease, which may have not been a large enough sample to evaluate heterogeneity of responses in relation to different phenotypes and disease severities associated with Crohn’s disease. In addition, Crohn’s disease patients had a median disease duration of 10 years, and we did not have a large enough sample to determine whether patient preferences differ among patients facing treatment decisions at the time of diagnosis. This study was conducted in Canada and should be replicated in other regions to determine the external validity of patient preferences. Finally, while we included several measures of internal validity, other tests exist [15] but were not included in the design of our DCE.

With the explosion of new therapeutic choices for the treatment of Crohn’s disease, these data serve to support patient and physician decision-making. Choices in prescribing treatment options for Crohn’s disease should consider preference of the patient, recognizing that most patients are motivated by efficacy, whereas a subset will make decisions based on avoiding potential risks. Physicians can tailor treatment options based on patient preferences.

## Supporting information

S1 Fig Screenshots of discrete-choice experiment. (DOCX)

S1 Text JAGS model for discrete-choice experiment. (DOCX)

S1 Table Detailed discrete-choice experiment results for the main analysis (overall group). (DOCX)

S2 Table Univariate association between patient characteristics and 2-group latent class membership. (DOCX)

S3 Table Treatment comparisons between all treatments, accounting for both NMA outcomes and patient preferences. (DOCX)

S4 Table Pairwise treatments comparisons accounting for both NMA outcomes and patient preferences: Sensitivity Analysis #1—Biologic treatments assumed to not require a “Short course of prednisone initially”. (DOCX)

S5 Table Pairwise treatment comparisons accounting for both NMA outcomes and patient preferences: Sensitivity Analysis #2—Infliximab and adalimumab also assumed to also have a risk of “Possible low blood counts or liver reaction”. (DOCX)
