# Supplementary figures and images for: Patient preferences for maintenance therapy in Crohn’s disease: A discrete-choice experiment
Source: PLoS One. 2020 Jan 16;15(1):e0227635. doi: 10.1371/journal.pone.0227635 (PMC6964885; doi:10.1371/journal.pone.0227635)

**Supplementary Figure(s) S1. Screenshots of discrete-choice experiment**


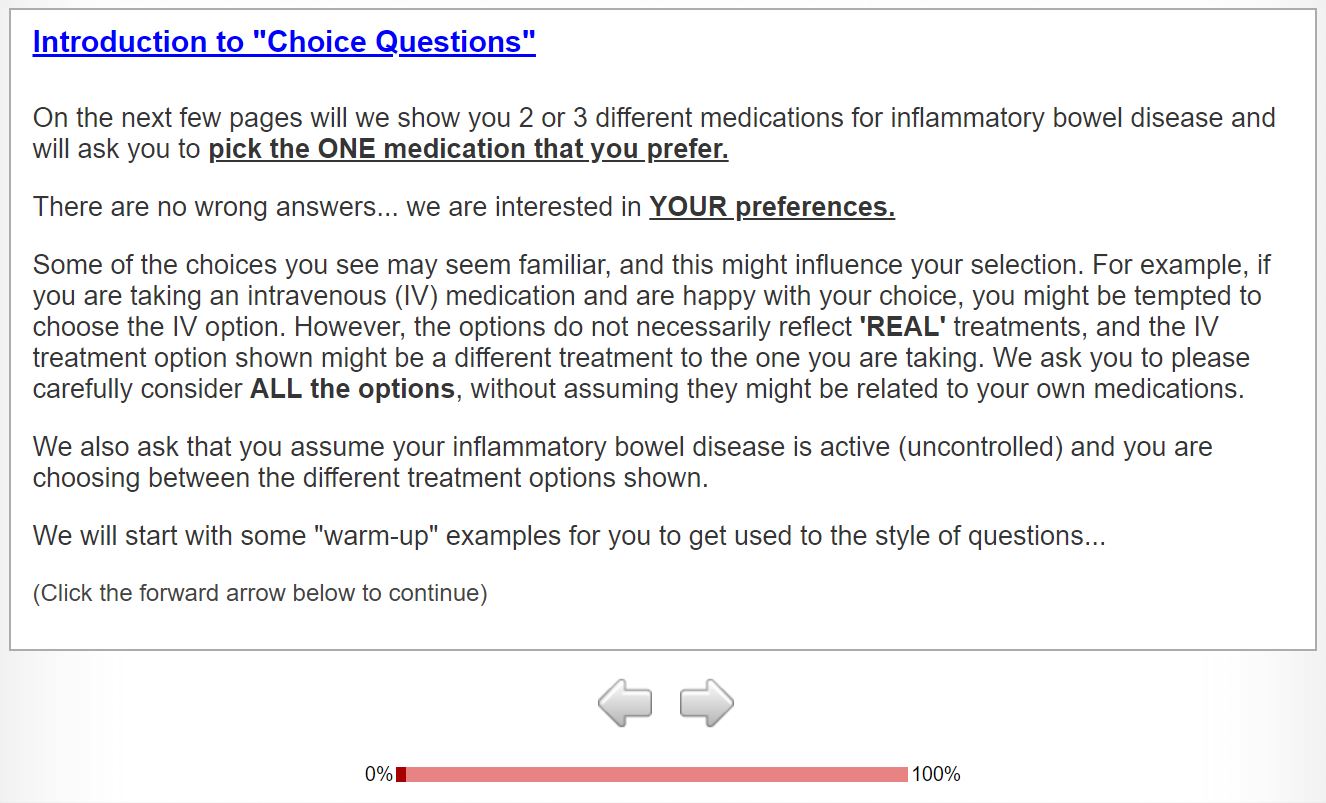


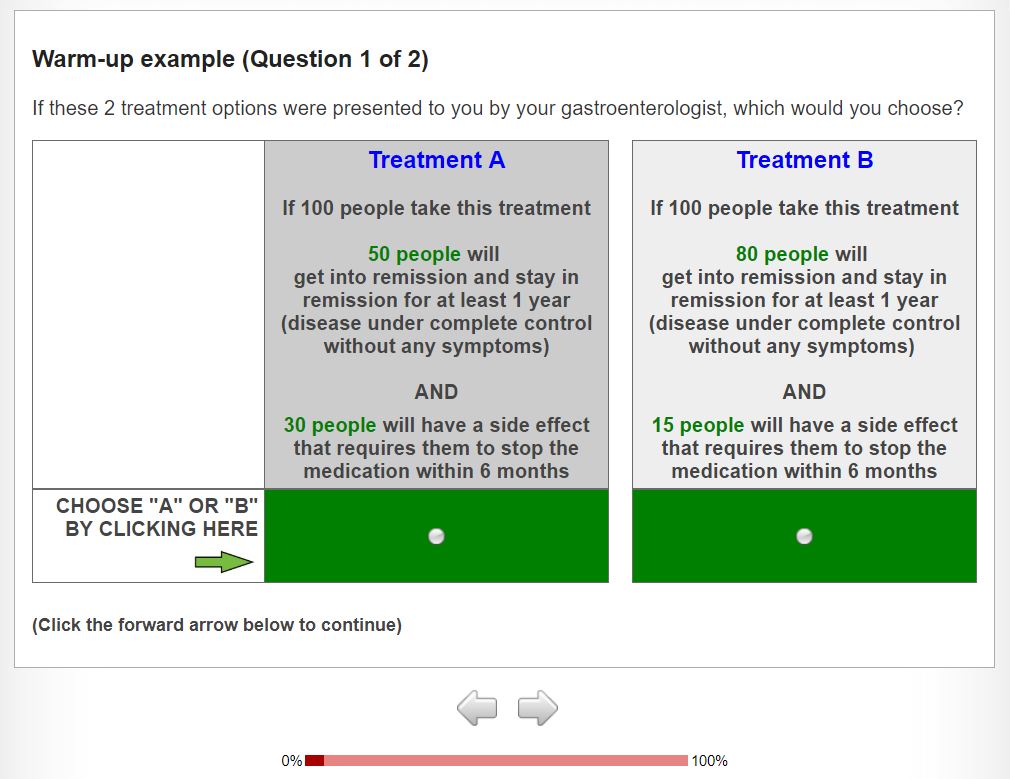


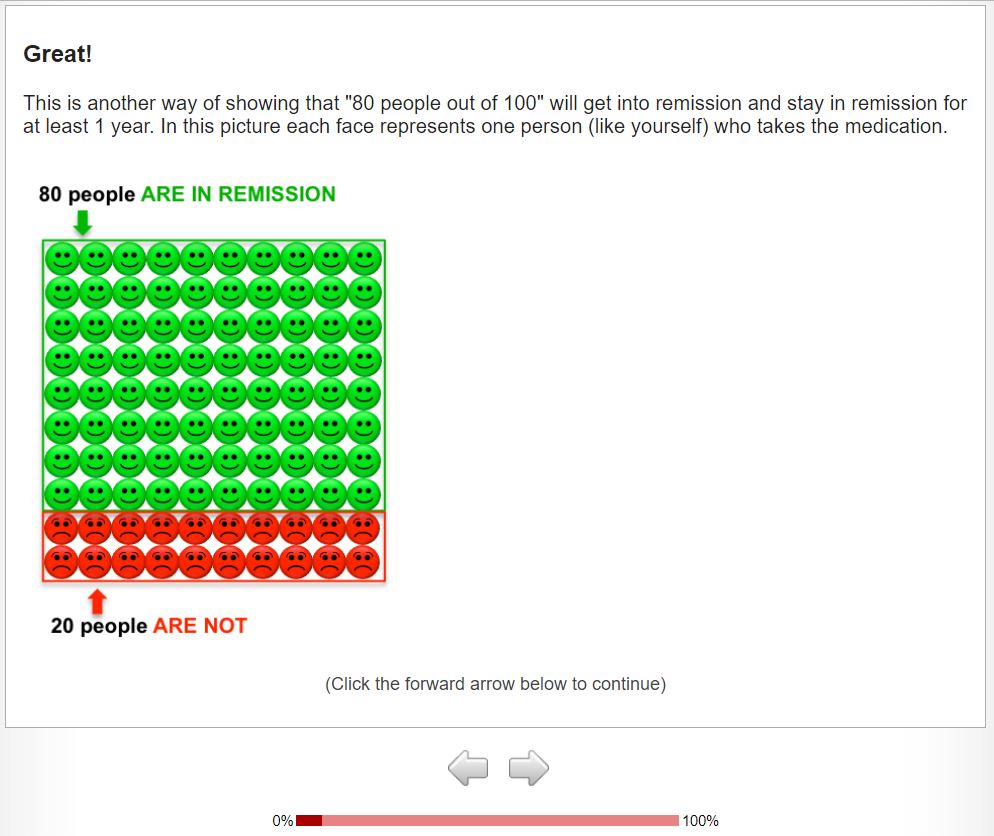


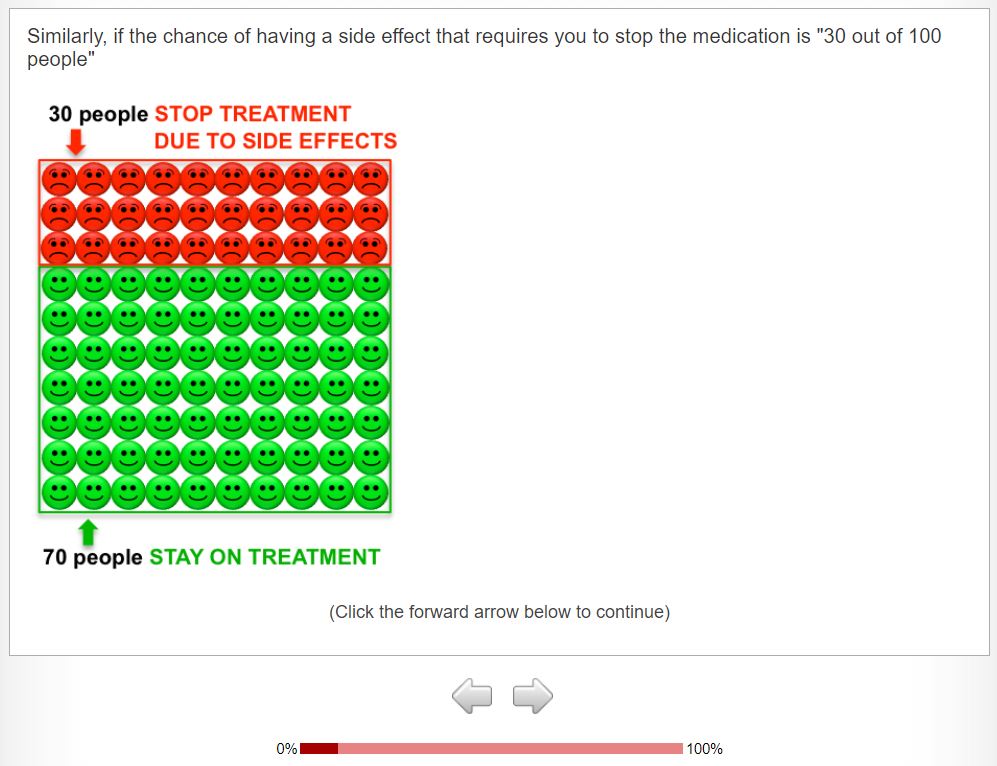


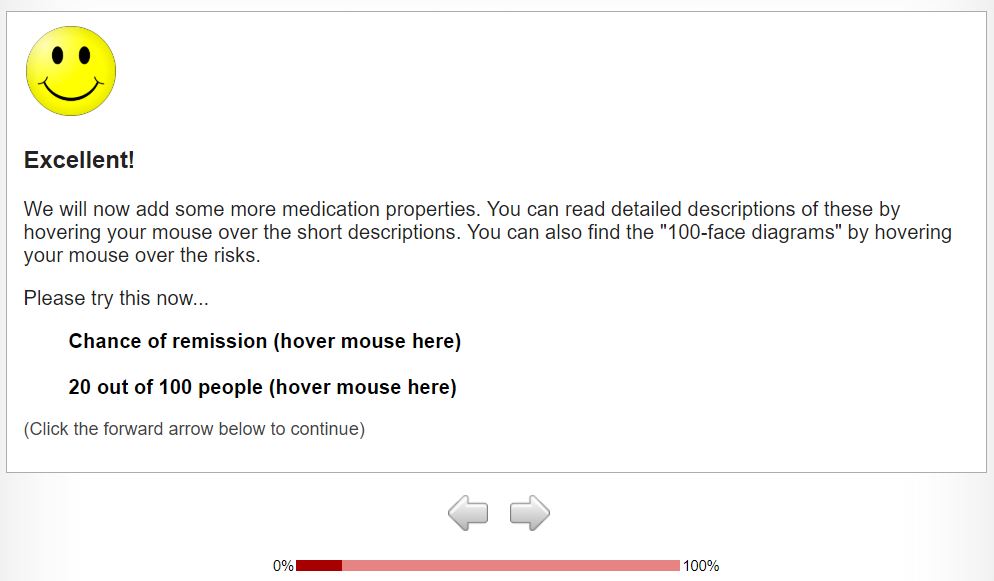


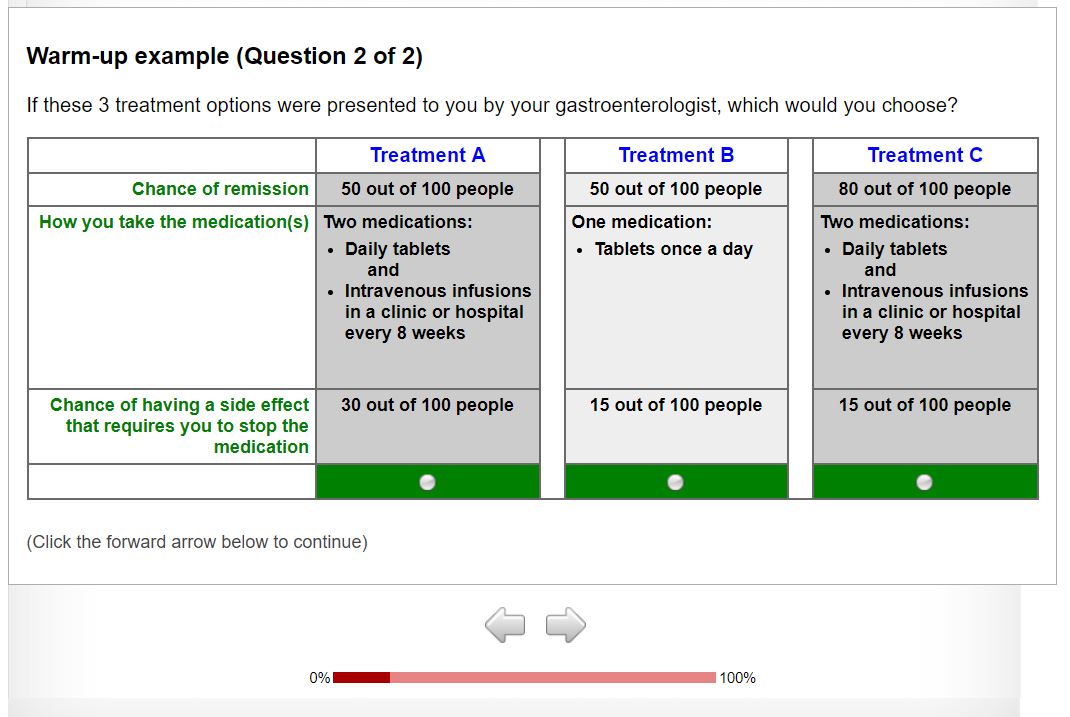


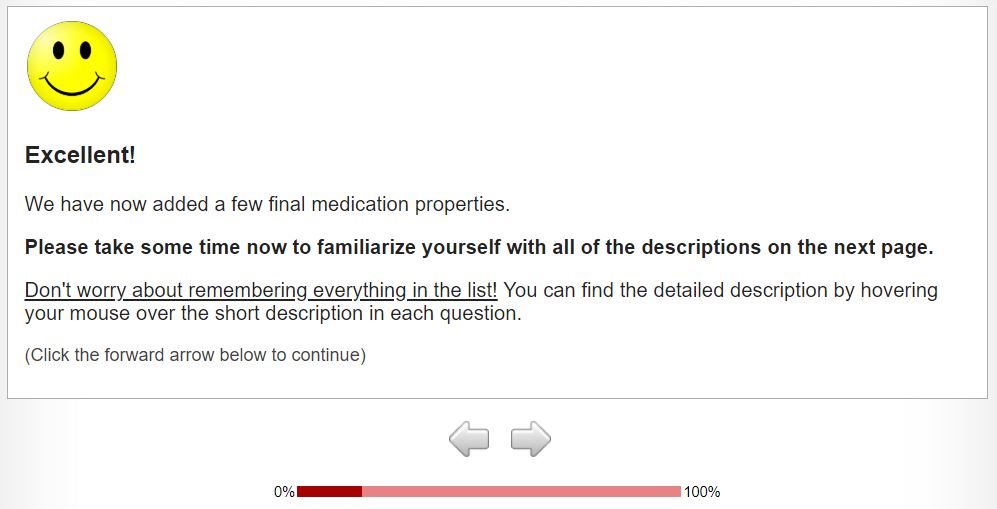


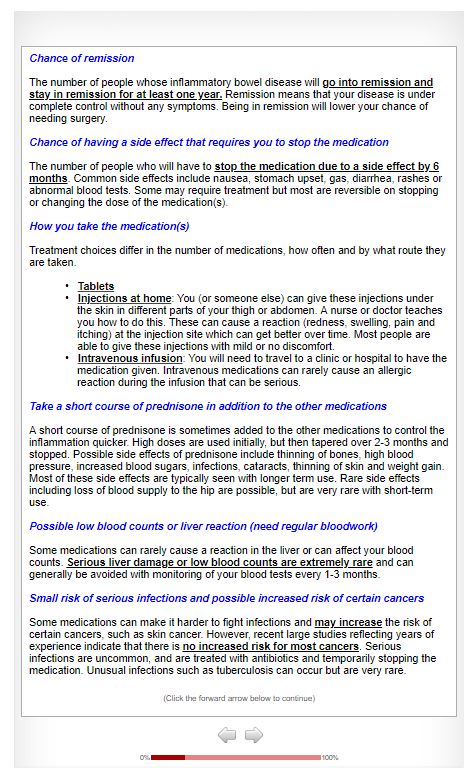


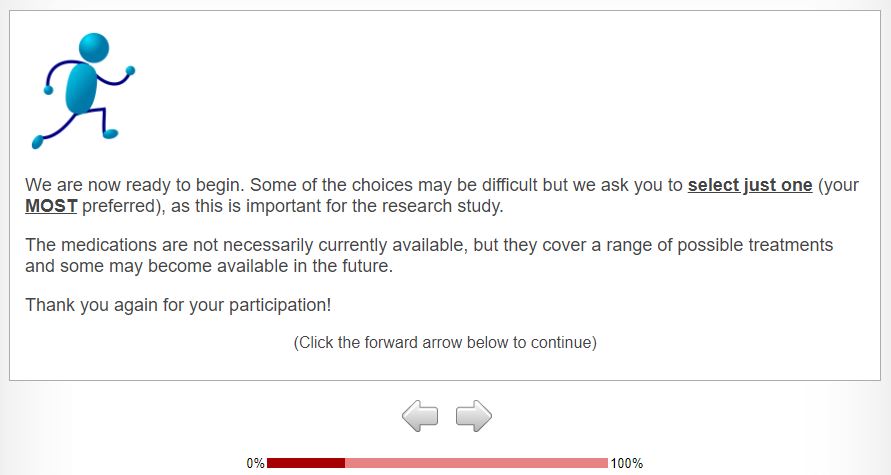


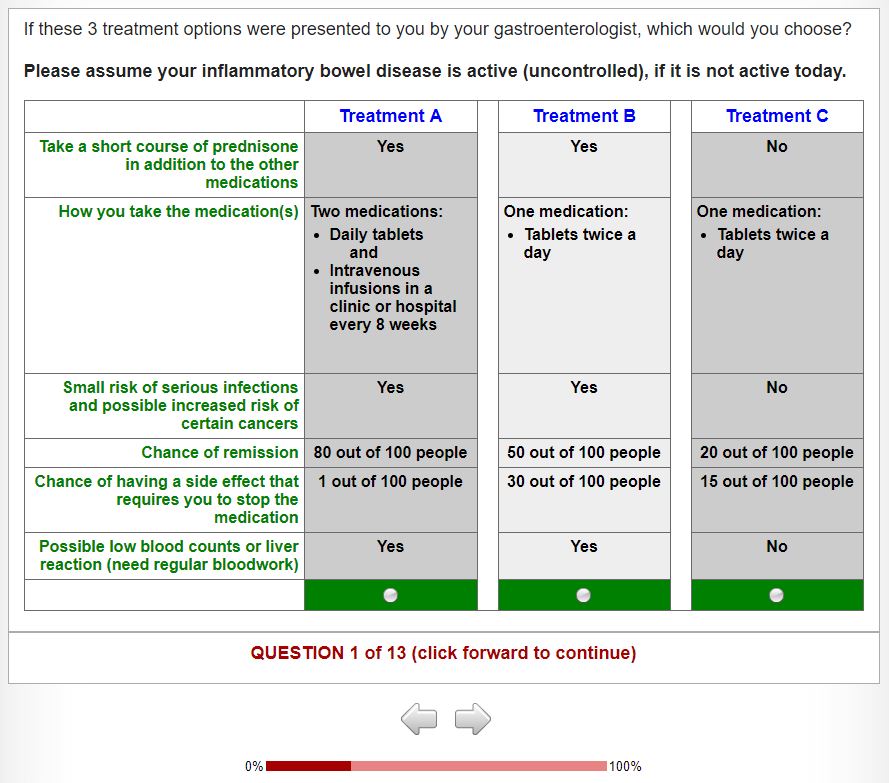

Supplement: S1 Fig — (DOCX) [file pone.0227635.s001.docx]
