# Supplementary material for: Patient preferences for maintenance therapy in Crohn’s disease: A discrete-choice experiment
Source: PLoS One. 2020 Jan 16;15(1):e0227635. doi: 10.1371/journal.pone.0227635 (PMC6964885; doi:10.1371/journal.pone.0227635)
Supplement: S1 Text — (DOCX) [file pone.0227635.s002.docx]

**Supplementary Text 1 - JAGS model for discrete-choice experiment**

model{
#Likelihood

for(i in 1:Nexp){

Y[i]~dcat(p[i,1:Nchoice])

}

#Link

for(i in 1:Nexp){

sumeprod[i] <- sum(e.util[i,])

for(s in 1:Nchoice){

p[i,s] <- e.util[i,s]/sumeprod[i]

e.util[i,s] <- exp(util[i,s])

#Regression

util[i,s] <- beta[ID[i],1]*remission[i,s]

+ beta[ID[i],2]*wdtox[i,s]

+ beta[ID[i],3]*dosing.1[i,s]

+ beta[ID[i],4]*dosing.2[i,s]

+ beta[ID[i],5]*dosing.3[i,s]

+ beta[ID[i],6]*dosing.4[i,s]

+ beta[ID[i],7]*dosing.5[i,s]

+ beta[ID[i],8]*liver[i,s]

+ beta[ID[i],9]*pred[i,s]

+ beta[ID[i],10]*cancer[i,s]

}

}

#Priors
for(j in 1:Npeople){

beta[j, 1:nvar] ~ dmnorm(B[1:nvar], PREC[1:nvar,1:nvar])
}

#Prior on between-study precision matrix tau

#R is "mean" inverse precision

#20 is the 'scale' df

PREC[1:nvar,1:nvar] ~ dwish(R[1:nvar,1:nvar],20)

#priors on population mean tmt effects

B[1:nvar] ~ dmnorm(mn[1:nvar],P[1:nvar,1:nvar])

}
