# Supplementary material for: Patient preferences for maintenance therapy in Crohn’s disease: A discrete-choice experiment
Source: PLoS One. 2020 Jan 16;15(1):e0227635. doi: 10.1371/journal.pone.0227635 (PMC6964885; doi:10.1371/journal.pone.0227635)
Supplement: S1 Table — (DOCX) [file pone.0227635.s003.docx]

**Table S1: Detailed DCE results for the main analysis (overall group)**

| Attribute | Levels | Utility  Median (CrI) | Relative importance  Median (CrI) | Marginal rate of substitution  (relative to remission)  Median (CrI) |
| --- | --- | --- | --- | --- |
| Remission | 20% to 80% | 10 (8.69, 11.47) | 45.89 (39.51, 52.27) | -- |
| Withdrawal due to AE | 1% to 30% | -4.07(-5.06, -3.25) | 18.68 (14.53, 22.83) | 24.42 (19, 29.85) |
| Dosing regime | One med: daily pills | 0.69 (0.21, 1.19) | 8.35 (7.57, 9.13) | 10.92 (9.9, 11.94) |
|  | One med: pills BID | -0.04 (-0.58, 0.47) |  |  |
|  | One med: Weekly sc | 0.26 (-0.28,0.80) |  |  |
|  | One med: Q2W sc | 0.27 (-0.30, 0.85) |  |  |
|  | One med: Q8W IV | -0.05 (-0.62,0.52) |  |  |
|  | Two meds: Q8W IV + daily pills | -1.13 (-1.80, -0.48) |  |  |
| Prednisone | Yes | -1.14 (-1.45, -0.85) | 10.46 (9.09, 11.84) | 13.68 (11.88, 15.48) |
|  | No | 1.14 (0.85, 1.45) |  |  |
| Infection/possible increased risk cancer | Yes | -1.20 (-1.53, -0.89) | 11.01 (9.55, 12.48) | 14.4 (12.49, 16.32) |
|  | No | 1.20 (0.89, 1.53) |  |  |
| Low blood counts/liver reaction | Yes | -0.61 (-0.85, -0.38) | 5.6 (4.52, 6.68) | 7.32 (5.91, 8.73) |
|  | No | 0.61 (0.38, 0.85) |  |  |

AE, adverse event; BID, twice daily; CrI, credible interval; IV, intravenous; med, medication; sc, subcutaneous; Q2W, every 2 weeks; Q8W, every 8 weeks
