# Supplementary material for: Patient preferences for maintenance therapy in Crohn’s disease: A discrete-choice experiment
Source: PLoS One. 2020 Jan 16;15(1):e0227635. doi: 10.1371/journal.pone.0227635 (PMC6964885; doi:10.1371/journal.pone.0227635)
Supplement: S2 Table — (DOCX) [file pone.0227635.s004.docx]

**Table S2: Univariate association between patient characteristics and 2-group latent class membership**

| Patient  characteristics | Risk  tolerant group  (n=84) | Risk  averse  group  (n=71) | Odds ratio for membership in risk averse group  (95% CI) | *P*-value |
| --- | --- | --- | --- | --- |
| Age, years, median (IQR) | 40 (21) | 40 (22) | 1.00 (0.98, 1.02) | 0.691 |
| Female, n (%) | 55 (65) | 51 (72) | 1.34 (0.68, 2.67) | 0.397 |
| Current or former Smoker, n (%) | 19 (23) | 19 (27) | 1.25 (0.60, 1.60) | 0.551 |
| Perianal disease, n (%) | 19 (23) | 18 (25) | 1.16 (0.55, 1.43) | 0.691 |
| Prior abdominal surgery, n (%) | 34 (40) | 29 (41) | 1.02 (0.53, 1.93) | 0.962 |
| Disease duration, years, median (IQR) | 10 (15) | 9 (12) | 0.98 (0.96, 1.02) | 0.494 |
| **Prior treatment** |  |  |  |  |
| Azathioprine, n (%) | 61 (73) | 62 (87) | 2.60 (1.11, 6.06) | 0.027 |
| Corticosteroids, n (%) | 46 (55) | 49 (69) | 1.83 (0.95, 3.56) | 0.071 |
| Infliximab, n (%) | 39 (46) | 30 (42) | 0.84 (0.45, 1.60) | 0.602 |
| Adalimumab, n (%) | 44 (52) | 34 (49) | 0.85 (0.44, 1.60) | 0.577 |
| Methotrexate, n (%) | 28 (33) | 20 (28) | 0.78 (0.39, 1.56) | 0.488 |
| Vedolizumab, n (%) | 4 (5) | 2 (3) | 0.58 (0.10, 3.26) | 0.536 |

CI, confidence interval; IQR, interquartile range
