# Supplementary material for: Patient preferences for maintenance therapy in Crohn’s disease: A discrete-choice experiment
Source: PLoS One. 2020 Jan 16;15(1):e0227635. doi: 10.1371/journal.pone.0227635 (PMC6964885; doi:10.1371/journal.pone.0227635)
Supplement: S3 Table — (DOCX) [file pone.0227635.s005.docx]

**Table S3. Treatment comparisons between all treatments, accounting for both NMA outcomes and patient preferences**

|  | Probability that the treatment is preferred (%) | | |
| --- | --- | --- | --- |
| Intervention | Main analysis  (base case) | Sensitivity analysis #1  (no prednisone for biologic therapy) | Sensitivity analysis #2 (infliximab/adalimumab also have a risk of low blood counts/ liver reaction) |
| Azathioprine | 0.9 | 0.3 | 1.1 |
| Infliximab | 2.1 | 2.1 | 0.6 |
| Infliximab + azathioprine | 13.7 | 13.7 | 16.2 |
| Vedolizumab | 30.2 | 30.5 | 39.8 |
| Adalimumab | 52.9 | 53.2 | 41.9 |
| Methotrexate | 0.3 | 0.2 | 0.3 |
