# Supplementary material for: Patient preferences for maintenance therapy in Crohn’s disease: A discrete-choice experiment
Source: PLoS One. 2020 Jan 16;15(1):e0227635. doi: 10.1371/journal.pone.0227635 (PMC6964885; doi:10.1371/journal.pone.0227635)
Supplement: S4 Table — (DOCX) [file pone.0227635.s006.docx]

**Table S4. Pairwise treatments comparisons accounting for both NMA outcomes and patient preferences: Sensitivity Analysis #1 - Biologic treatments assumed to not require a “Short course of prednisone initially”**

|  | Comparator | | | | |
| --- | --- | --- | --- | --- | --- |
|  | (probability the intervention is better than the comparator) | | | | |
| Intervention | Azathioprine | Infliximab | Infliximab + azathioprine | Vedolizumab | Adalimumab |
| Infliximab | 92% |  |  |  |  |
| Infliximab + azathioprine | 92% | 53% |  |  |  |
| Vedolizumab | 97% | 71% | 70% |  |  |
| Adalimumab | 98% | 81% | 79% | 64% |  |
| Methotrexate | 38% | 5% | 5% | 2% | 1% |
