# Supplementary material for: Patient preferences for maintenance therapy in Crohn’s disease: A discrete-choice experiment
Source: PLoS One. 2020 Jan 16;15(1):e0227635. doi: 10.1371/journal.pone.0227635 (PMC6964885; doi:10.1371/journal.pone.0227635)
Supplement: S5 Table — (DOCX) [file pone.0227635.s007.docx]

**Table S5. Pairwise treatment comparisons accounting for both NMA outcomes and patient preferences: Sensitivity Analysis #2 - Infliximab and adalimumab also assumed to also have a risk of “Possible low blood counts or liver reaction”**

|  | Comparator | | | | |
| --- | --- | --- | --- | --- | --- |
|  | (probability the intervention is better than the comparator) | | | | |
| Intervention | Azathioprine | Infliximab | Infliximab + azathioprine | Vedolizumab | Adalimumab |
| Infliximab | 70% | -- | -- | -- | -- |
| Infliximab + azathioprine | 79% | 65% | -- | -- | -- |
| Vedolizumab | 91% | 80% | 70% | -- | -- |
| Adalimumab | 90% | 81% | 70% | 53% | -- |
| Methotrexate | 38% | 18% | 12% | 5% | 6% |
